# P2Y_2_R activation by nucleotides released from the highly metastatic breast cancer cell contributes to pre-metastatic niche formation by mediating lysyl oxidase secretion, collagen crosslinking, and monocyte recruitment

**DOI:** 10.18632/oncotarget.2427

**Published:** 2014-09-02

**Authors:** Young Nak Joo, Hana Jin, So Young Eun, Sang Won Park, Ki Churl Chang, Hye Jung Kim

**Affiliations:** ^1^ Department of Pharmacology, School of Medicine, Institute of Health Sciences, Gyeongsang National University, Jinju, Korea

**Keywords:** collagen crosslinking, HIF-1α, LOX, nucleotides, premetastatic niche formation, P2Y_2_R

## Abstract

Tumor microenvironmental hypoxia induces hypoxia inducible factor-1α (HIF-1α) overexpression, leading to the release of lysyl oxidase (LOX), which crosslinks collagen at distant sites to facilitate environmental changes that allow cancer cells to easily metastasize. Our previous study showed that activation of the P2Y_2_ receptor (P2Y_2_R) by ATP released from MDA-MB-231 cells increased MDA-MB-231 cell invasion through endothelial cells. Therefore, in this study, we investigated the role of P2Y_2_R in breast cancer cell metastasis to distant sites. ATP or UTP released from hypoxia-treated MDA-MB-231 cells induced HIF-1α expression and LOX secretion by the activation of P2Y_2_R, and this phenomenon was significantly reduced in P2Y_2_R-depleted MDA-MB-231 cells. Furthermore, P2Y_2_R-mediated LOX release induced collagen crosslinking in an *in vitro* model. Finally, nude mice injected with MDA-MB-231 cells showed high levels of LOX secretion, crosslinked collagen and CD11b^+^ BMDC recruitment in the lung; however, mice that were injected with P2Y_2_R-depleted MDA-MB-231 cells did not exhibit these changes. These results demonstrate that P2Y_2_R plays an important role in activation of the HIF-1α–LOX axis, the induction of collagen crosslinking and the recruitment of CD11b^+^ BMDCs. Furthermore, P2Y_2_R activation by nucleotides recruits THP-1 monocytes, resulting in primary tumor progression and pre-metastatic niche formation.

## INTRODUCTION

Breast cancer is an epithelial malignancy of the ducts or lobules within the breast. Breast cancer is one of the most common cancers diagnosed in women in North America and Western Europe [1,2]; although Asian populations generally have the lowest risk, rates in this population have been steadily increasing. Moreover, breast cancer is not limited to women and is diagnosed in 2,000 men per year in the United States [3]. Cancer metastasis, the process by which cancer cells spread from the site of origin to grow in adjacent sites, poses the greatest challenge to cancer treatment, and metastasis, rather than the primary tumor, is responsible for the majority of cancer deaths [4-6]. Metastasis also contributes to over 90% of breast cancer patient deaths. The 10-year survival rate for patients with metastases at distant sites such as the lung, bone, and brain is 9%, which is significantly lower than the 56% survival rate for patients with metastases at local sites near the primary tumor [3,7]. For these reasons, studies have focused on validating the molecular mechanisms of distant metastasis.

During metastasis, gene expression signature changes prevent cell death mechanisms from disrupting cell-cell or cell-matrix interactions and result in cancer cells that are resistant to death [8]. Moreover, these changes in gene expression can stimulate preferential cancer cell metastasis to particular organs. However, receptive microenvironments for cancer cell establishment are a prerequisite for metastasis to distant sites. The tumor microenvironment is populated by many types of cells, including adipocytes, fibroblasts, a wide range of hematopoietic cells, macrophages, and neutrophils, as well as newly formed blood and lymphatic vessels [9]. Within the tumor microenvironment, tumors release extracellular signals such as vascular endothelial growth factor (VEGF), matrix metalloproteinases (MMPs), lysyl oxidase (LOX), and nucleotides to survive. Macrophages localized within the tumor, termed tumor-associated macrophages (TAMs), can also release various growth factors, MMPs, and cytokines including VEGF, TNF-α, and thymidine phosphorylase that stimulate cancer metastasis [10-12] and have been shown to correlate with a poor prognosis in breast cancer [13]. Moreover, co-culture of TAMs with breast cancer cells upregulates the release of cytokines and MMPs by the macrophages [14]. With increasing tumor mass, hypoxia often results from insufficient blood supply to the cancer cells, and tumor microenvironmental hypoxia induces increased expression of hypoxia inducible factor-1α (HIF-1α), leading to the release of LOX, an enzyme that crosslinks extracellular matrix proteins such as collagen and promotes breast cancer metastasis. Clinical studies have shown that both HIF-1α and LOX are overexpressed in breast cancer patients, and this overexpression increases with disease progression, resulting in a high mortality rate [15,16].

Recently, a model termed ‘pre-metastatic niche formation’ was proposed to understand the distant metastasis of cancer. A pre-metastatic niche is defined as a fertile microenvironment that forms in a metastatic target organ and facilitates the invasion, survival, and/or proliferation of metastatic tumor cells, providing a novel mechanism for the promotion of metastasis [17]. Prior recruitment of hematopoietic bone marrow-derived cells (BMDCs) to crosslinked collagen at distant sites facilitates environmental changes that promote cancer cell colonization and metastasis [18-20].

As mentioned above, tumor cells and TAMs release nucleotides. In particular, ATP is released and accumulates at a much higher concentration in the tumor interstitium than in healthy tissues [21], and recent reports have highlighted the involvement of ATP in tumor progression. Our previous results also showed that the highly metastatic breast cancer cell line MDA-MD-231 released significantly more ATP than the less metastatic breast cancer cell line MCF-7 or normal epithelial or endothelial cells (ECs) [22]. Moreover, we showed that ATP released from MDA-MB-231 cells increased proliferation, migration, and invasion through ECs by activating the P2Y_2_ receptor (P2Y_2_R) [22]. P2Y_2_R is a G protein-coupled purinergic receptor activated by both extracellular ATP and UTP [23]. Many studies have shown that extracellular purines accumulate in the tumor microenvironment and directly affect cancer progression through purinergic receptors. Activation of P2Y_2_Rs has also been shown to support the progression of each step of metastasis, including angiogenesis, intravasation, and invasion, as well as tumor growth [24-26]. Therefore, it has been speculated that P2Y_2_R might be involved in metastatic seeding at secondary sites. However, the precise mechanisms for how P2Y_2_R contributes to the ‘pre-metastatic niche formation’ model of distant metastasis are not yet understood. Thus, in the present study, we investigated whether P2Y_2_R plays an important role in pre-metastatic niche formation, specifically in the recruitment of hematopoietic BMDCs to crosslinked collagen located at distant sites through activation of the HIF-1α-LOX axis.

## RESULTS

### Nucleotides released in response to hypoxia induce HIF-1α expression in MDA-MB-231 cells, but not in MCF-7 cells, through the activation of P2Y_2_R

**Fig.1:**
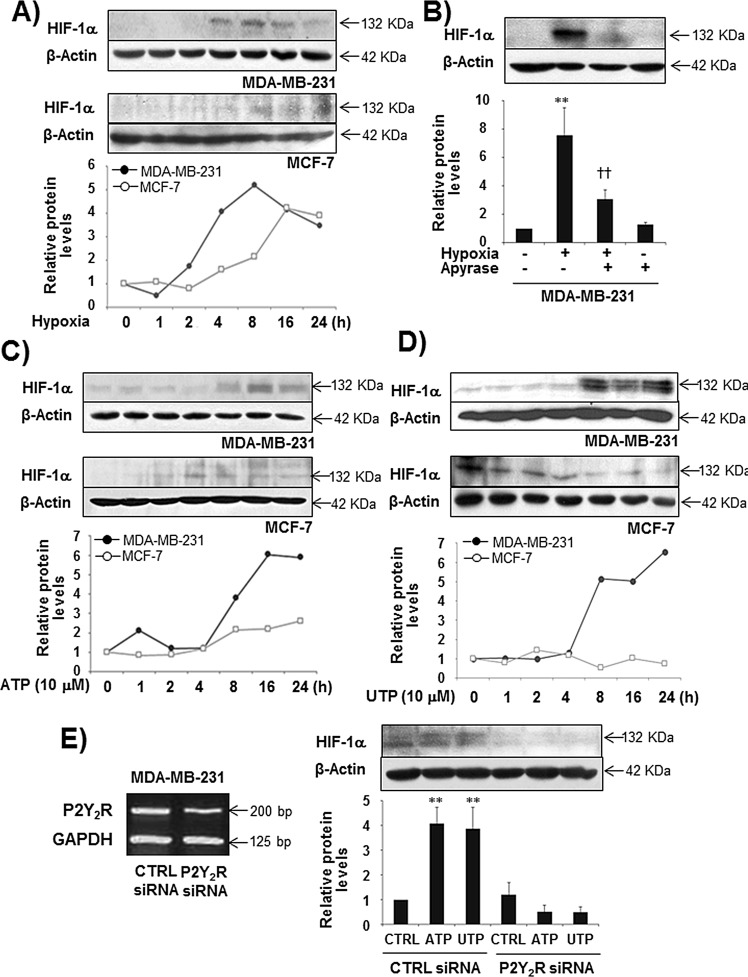
Hypoxia and ATP/UTP induced HIF-1α expression through the activation of P2Y_2_R in MDA-MB-231 but not MCF-7 cells (A) MDA-MB-231 and MCF-7 cells were exposed to hypoxia (2% O_2_) for various time periods (1 - 24 h), and HIF-1α expression was determined from cell lysates by Western blotting as described in the Methods. (B) MDA-MB-231 cells were pretreated with or without apyrase for 1 h and then exposed to hypoxia for 8 h. HIF-1α expression levels were determined as described above. Data represent mean values ± SEM of three independent experiments (compared with the control, **P < 0.01; compared with hypoxia, ††*P* < 0.01). (C, D) MDA-MB-231 and MCF-7 cells were treated with ATP (10 μM) or UTP (10 μM) in a time-dependent manner (1 - 24 h), and HIF-1α expression levels were determined as above. (E) Control siRNA- or P2Y_2_R siRNA-transfected MDA-MB-231 cells were treated with ATP or UTP (10 μM) for 8 h, and HIF-1α protein levels were determined by Western blot analysis. Data represent mean values ± SEM of three independent experiments (compared with control, **P < 0.01, *P < 0.05).

Extracellular nucleotides are released and detectable at high concentrations within the tumor microenvironment. In our previous study [22], the highly metastatic breast cancer cell line MDA-MB-231 released ATP at a much higher level than the less metastatic breast cancer cell line MCF-7, although P2Y_2_R expression was not different between the two cell types. In addition, under hypoxia, MDA-MB-231 cells released more ATP compared with MCF-7 or the normal epithelial breast cell line MCF-10A (Supplemental Fig 1). Thus, we first investigated the role of P2Y_2_R in HIF-1α expression leading to pre-metastatic niche formation. We confirmed that hypoxia induces HIF-1α expression in both MDA-MB-231 and MCF-7 cells. The highly metastatic breast cancer cells MDA-MB-231 induced HIF-1α expression in a time-dependent manner with peak levels observed at 8 h, whereas the less metastatic breast cancer cells MCF-7 showed a weak induction of HIF-1α compared with MDA-MB-231 cells (Fig 1A). This induction of HIF-1α by MDA-MB-231 cells in response to hypoxia was abolished in the presence of apyrase (Fig 1B). Moreover, ATP or UTP (10 M) treatment significantly increased HIF-1α expression from 8 h until 24 h in MDA-MB-231 cells but not in MCF-7 cells (Fig 1C, D). P2Y_2_R-depleted MDA-MB-231 cells did not show induced HIF-1α expression (Fig 1E). These results suggest that nucleotides released from MDA-MB-231 cells exposed to hypoxia induce HIF-1α expression by activating P2Y_2_R.

### P2Y_2_R activation by ATP or UTP induces LOX secretion in MDA-MB-231 cells but not in MCF-7 cells

**Fig.2:**
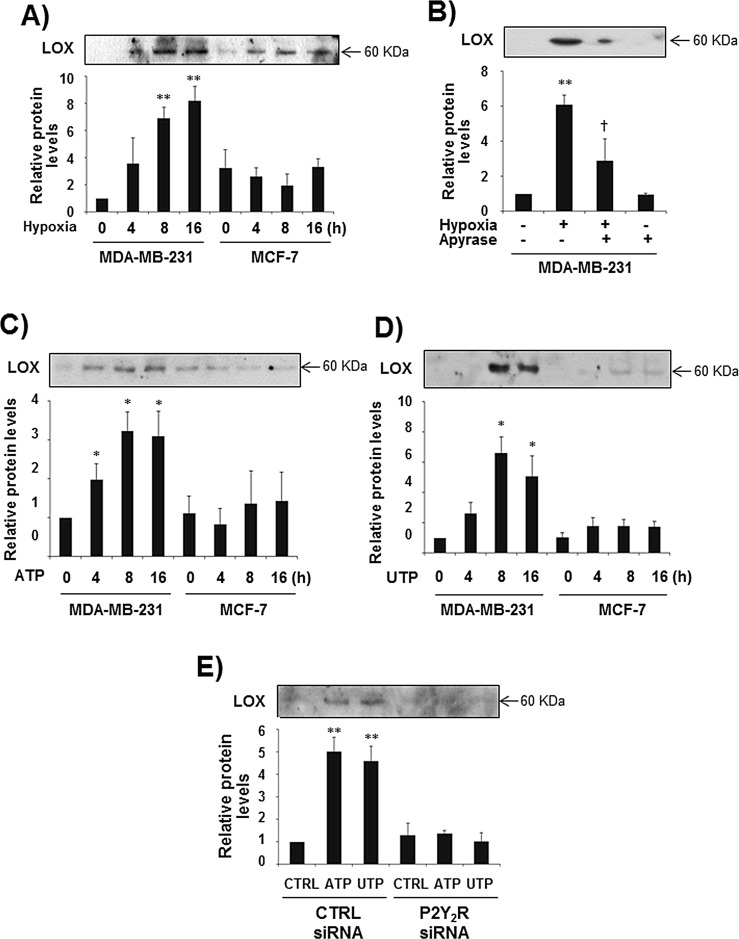
P2Y_2_R activation by ATP or UTP induced LOX secretion in MDA-MB-231 cells but not in MCF-7 cells (A) MDA-MB-231 and MCF-7 cells were exposed to hypoxia for 4, 8, and 16 h. After incubation for the indicated time, CM were collected and concentrated 20 times, and LOX levels in the CM were determined by Western blot analysis as described in the Methods. (B) MDA-MB-231 cells were pretreated with or without apyrase for 1 h and then exposed to hypoxia for 8 h. LOX levels from the CM were determined as described above. (C, D) MDA-MB-231 and MCF-7 cells were treated with ATP (10 μM) or UTP (10 μM) for 4, 8, and 16 h, and then LOX levels in the CM were determined by Western blotting as described above. (E) Control siRNA- or P2Y_2_R siRNA-transfected MDA-MB-231 cells were treated with ATP (10 μM) or UTP (10 μM) for 8 h, and LOX levels in the CM were determined as described above. Data represent mean values ± SEM of three independent experiments (compared with control, **P < 0.01, *P < 0.05).

It has been reported that hypoxia-induced expression of HIF-1α leads to the release of LOX, an enzyme that crosslinks extracellular matrix proteins such as collagen and promotes breast cancer metastasis [20]. Accordingly, we evaluated the role of P2Y_2_R in LOX secretion. As expected, hypoxia dramatically induced LOX release at 8 h and 16 h in MDA-MB-231 cells but not in MCF-7 cells, and hypoxia-induced LOX secretion was significantly reduced by apyrase in MDA-MB-231 cells (Fig 2A, B). In addition, treatment with ATP or UTP (10 μM) resulted in a significant induction of LOX release at 8 h and 16 h in control siRNA-transfected MDA-MB-231 cells but not in P2Y_2_R siRNA-transfected MDA-MB-231 cells (Fig 2C-E).

### P2Y_2_R-mediated LOX release leads to collagen crosslinking

**Fig.3:**
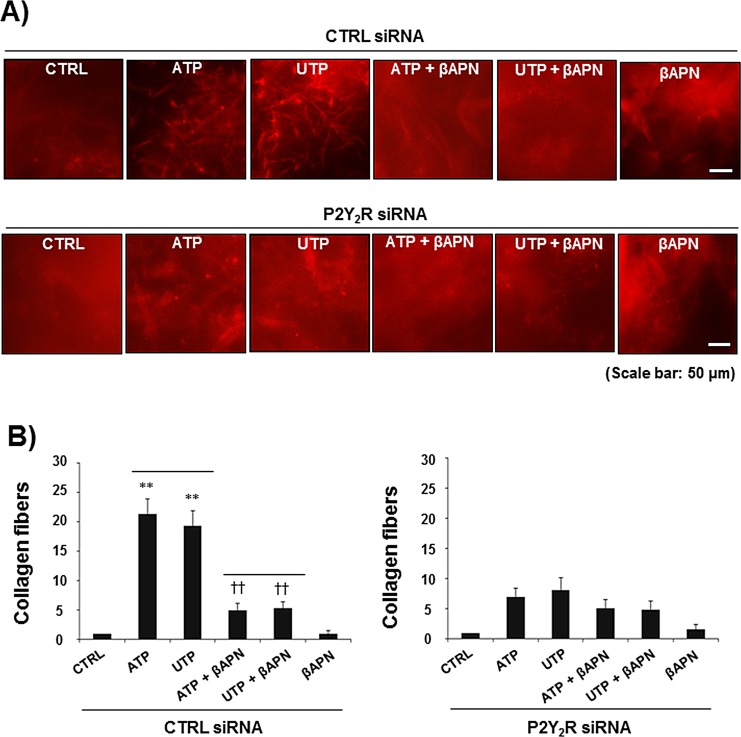
P2Y_2_R-mediated LOX release induced collagen crosslinking CTRL siRNA-transfected MDA-MB-231 cells (A) or P2Y_2_R siRNA-transfected MDA-MB-231 cells (B) were pretreated with βAPN (300 μM), a LOX inhibitor, for 1 h and then stimulated with ATP (10 μM) or UTP (10 μM) for 8 h. CM from the cells were concentrated 20-fold, and 100 μl of CM was mixed with collagen type I solution (4 mg/ml, 100 μl) as described in the Methods. After a 16-h incubation in a 37°C cell culture incubator, the amount of crosslinked collagen was determined using a fluorescence microscope. Data represent mean values ± SEM of three independent experiments in triplicate (compared with the control, **P < 0.01; compared with ATP or UTP treatment, ^††^*P* < 0.01).

Next, we assessed the effect of P2Y_2_R activation on LOX-induced collagen remodeling. MDA-MB-231 cells were treated with ATP or UTP (10 μM) for 8 h, and CM were then collected from MDA-MB-231 cells and mixed with type I collagen. Fig 3A shows that the CM collected from ATP- or UTP-treated MDA-MB-231 cells significantly increased the amount of crosslinked collagen compared with the CM from untreated MDA-MB-231 cells. Treatment with βAPN (300 μM), a LOX inhibitor, reduced collagen crosslinking, suggesting that P2Y_2_R-mediated LOX release results in collagen crosslinking. This result was confirmed using the P2Y_2_R knockdown system, in which ATP or UTP (10 μM) failed to crosslink collagen in P2Y_2_R-depleted MDA-MB-231 cells in the presence or absence of βAPN (Fig 3B).

### THP-1 monocytes induce P2Y_2_R expression in hypoxia, and P2Y_2_R activation by ATP or UTP induces THP-1 migration

**Fig.4:**
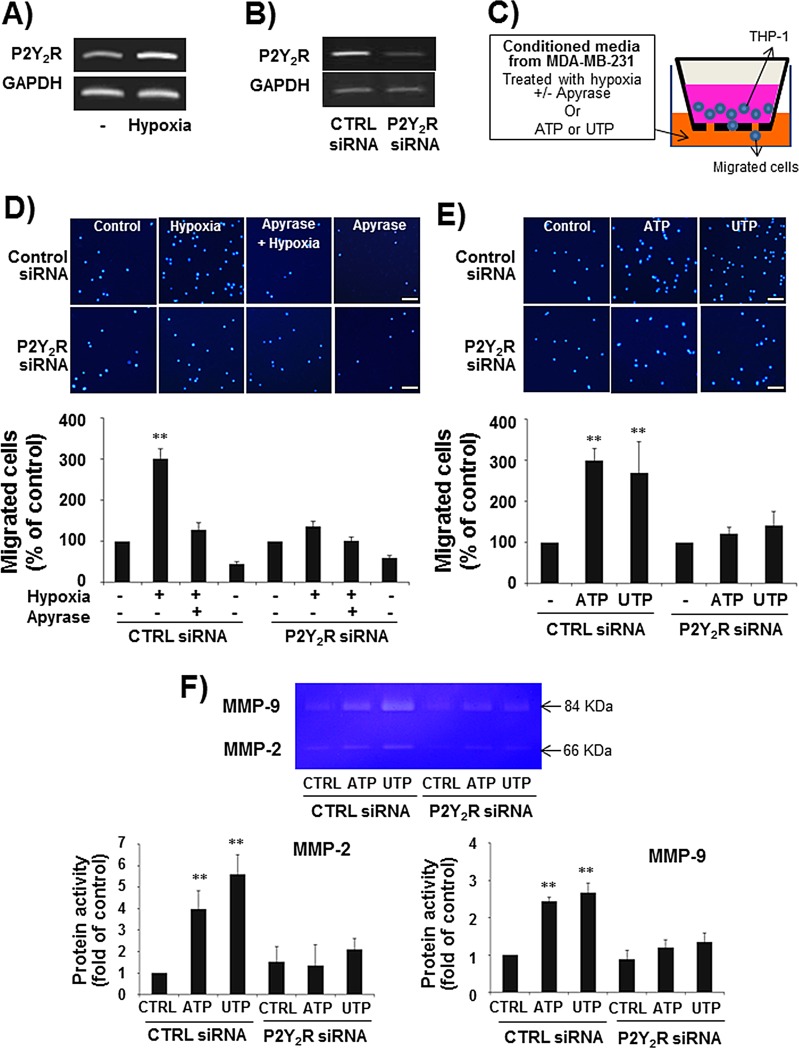
P2Y_2_R expression was increased in THP-1 human monocytes in hypoxia, and P2Y_2_R activation by ATP or UTP induced THP-1 migration and the release of MMPs A) THP-1 human monocytes were incubated in hypoxia for 24 h, and then P2Y_2_R mRNA was analyzed by RT-PCR. Results were confirmed by repeated experiments. B) THP-1 cells were transfected with control siRNA or P2Y_2_R siRNA (100 nM), and the efficiency of siRNA transfection was confirmed by RT-PCR. C-E) Control siRNA- or P2Y_2_R siRNA-transfected THP-1 cells (2 × 10^5^ cells) were added to 24-well cell culture inserts, and supernatants from MDA-MB-231 cells exposed to hypoxia in the presence or absence of apyrase (5 U/ml), an enzyme that rapidly hydrolyzes extracellular nucleotide 5′-diphosphates and triphosphates, for 8 h (D) or media containing ATP (10 μM) or UTP (10 μM) (E) were added to the lower chamber of the Transwell. After incubation for 6 h in a 37°C cell culture incubator, the cells that had migrated through the insert membranes were stained with DAPI and counted under a fluorescence microscope. Values represent the means ± SEM of 3 independent experiments (compared with the control, **P < 0.01, *P < 0.05) (scale bar: 50μM). F) Control siRNA- or P2Y_2_R siRNA-transfected THP-1 cells were treated with ATP (10 μM) or UTP (10 μM) for 24 h, and the activity of MMP-2 and MMP-9 in the CM was evaluated by gelatin zymography as described in the Methods.

When THP-1 human monocytes were exposed to hypoxia for 24 h, THP-1 cells induced P2Y_2_R expression as assessed by RT-PCR (Fig 4A). We then determined whether P2Y_2_R activation by ATP or UTP could induce THP-1 migration. First, to test whether ATP released from hypoxia-treated MDA-MB-231 cells induced THP-1 cell migration through P2Y_2_R activation, THP-1 cells were treated with CM from MDA-MB-231 cells exposed to hypoxia in the presence or absence of apyrase (Fig 4C). As shown in Fig 4D, CM from hypoxia-treated MDA-MB-231 cells induced THP-1 cell migration, whereas CM containing apyrase did not. To verify that nucleotides were capable of inducing the migration of THP-1 cells, ATP or UTP (10 μM) was added to the bottom chamber, and the migration of cells added to the upper chamber was measured. ATP and UTP increased cell migration by approximately 3- and 2.8-fold, respectively (Fig 4E). Moreover, P2Y_2_R siRNA-transfected THP-1 cells failed to migrate in response to CM from hypoxia-treated MDA-MB-231 cells or ATP or UTP treatment, suggesting that nucleotides from hypoxia-treated MDA-MB-231 cells induced THP-1 cells migration through P2Y_2_R activation in the THP-1 cells (Fig 4D, E). In addition, ATP or UTP (10 μM) treatment increased MMP activity, particularly that of MMP-9, in control siRNA-transfected THP-1 cells but not in P2Y_2_R siRNA-transfected THP-1 cells (Fig 4F).

### P2Y_2_R is involved in LOX secretion, collagen crosslinking, and BMDC recruitment to the lungs in an *in vivo* mouse model

**Fig.5:**
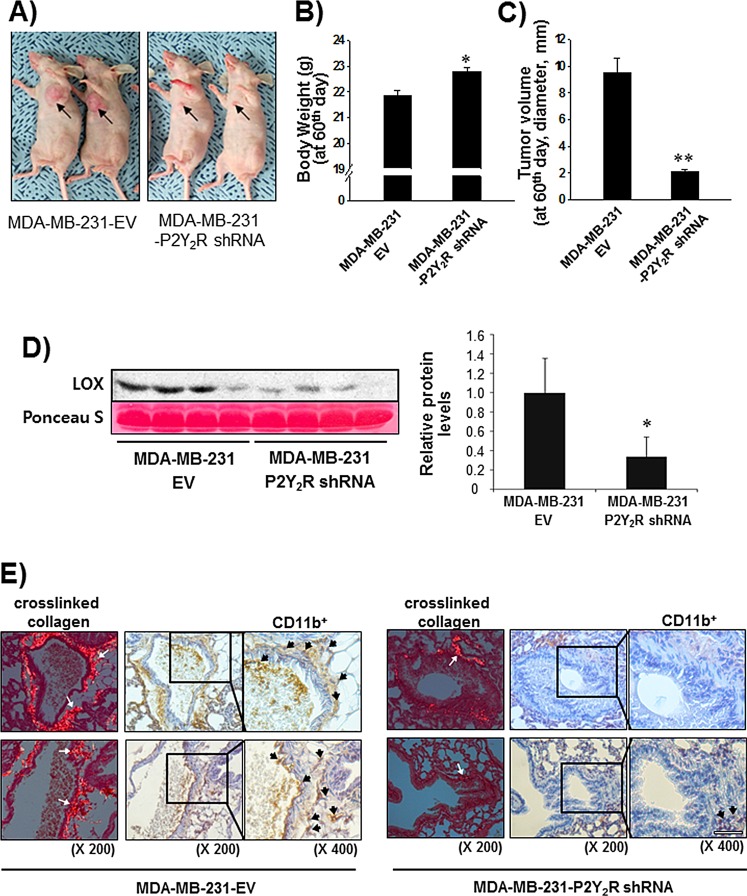
Inhibition of P2Y_2_R reduced collagen crosslinking and LOX secretion in an *in vivo* mouse model A) Athymic nude mice were divided into 2 groups and injected subcutaneously with MDA-MB-231-EV (n = 10) or MDA-MB-231-P2Y_2_R-shRNA (n = 10) (3 × 10^6^ cells/100 μl of serum-free medium). MDA-MB-231-EV-injected or MDA-MB-231- P2Y_2_R-shRNA-injected animals were sacrificed at day 60, and blood was collected by heart puncture. Body weights and tumor volumes were measured every 3 days during tumor development. Body weight (B) and tumor volume (C) at the end of 60 days are presented (compared with the MDA-MB-231-EV-injected mice, *P < 0.05). D) LOX levels in blood serum were analyzed by Western blot analysis (compared with the MDA-MB-231-EV-injected mice, *P < 0.05). E) Lung tissue sections from MDA-MB-231-EV- and MDA-MB-231-P2Y_2_R-shRNA-injected mice were stained with Picrosirius Red to analyze crosslinked collagen. Recruited CD11b^+^-immunoreactive BMDCs near the sites of collagen cross-linkage were counterstained with anti-CD11b antibody. White arrows indicate representative crosslinked collagen fibers. Black arrows indicate recruited CD11b^+^ BMDCs (scale bar: 50 μm).

Finally, we confirmed the role of P2Y_2_R in LOX-mediated collagen crosslinking and CD11b^+^ BMDC recruitment to the lung using an *in vivo* animal model. Nude mice were divided into two groups; mice in one group were subcutaneously injected with control-shRNA-transfected MDA-MB-231 cells (MDA-MB-231-EV), whereas mice in the other group were injected with P2Y_2_R-shRNA-transfected MDA-MB-231 cells (MDA-MB-231-P2Y_2_R-shRNA). Tumor volumes and body weights were measured every 3 days for 60 days. When the mice were sacrificed at the end of 60 days, body weight was significantly increased and tumor volume was decreased in the mice injected with MDA-MB-231-P2Y_2_R-shRNA compared to the mice injected with MDA-MB-231-EV (Fig 5A-C). Fig 5D shows that the LOX levels in the blood serum of mice injected with MDA-MB-231-P2Y_2_R-shRNA were significantly higher than those from MDA-MB-231-EV-injected mice. Moreover, crosslinked collagen was highly detected in lung sections from MDA-MB-231-EV-injected mice but not those of mice injected with MDA-MB-231-P2Y_2_R-shRNA. Additionally, MDA-MB-231-P2Y_2_R-shRNA-injected mice showed a lower level of CD11b^+^ BMDC recruitment around the sites of crosslinked collagen in the lungs compared to MDA-MB-231-EV-injected mice (Fig 5E).

## DISCUSSION

Virtually all patients who die from cancer, including breast cancer, have metastatic disease. Metastasis is a complex multistep process by which tumor cells acquire a specific and aggressive phenotype that allows them to escape from the primary tumor site. These cancer cells colonize organs with microenvironments that are advantageous and favorable for metastatic growth, and tumor and host cells within the tumor microenvironment coevolve and both play a key role in metastasis. ‘Pre-metastatic niche formation’ has become a representative model for secondary tumor growth at distant sites. Recently, it was found that LOX plays an important role in formation of the pre-metastatic niche [18]. LOX elevation occurs in metastatic and/or invasive breast cancer cell lines [27], and hypoxic primary tumor cells increase LOX expression and secretion, enabling cell movement to more oxygenated and thus more nutrient-rich areas. Mechanistically, LOX secreted by hypoxic tumor cells colocalizes with fibronectin at sites of future metastases and crosslinks collagen in the basement membrane, thereby permitting BMDC cell recruitment. Therefore, it is likely that the initial deposition of LOX in the pre-metastatic niche contributes to the generation of a suitable extracellular matrix facilitating the recruitment of BMDCs [28,29]. According to Wong et al. [20], HIF-1α overexpression induced the release of LOX into the bloodstream, which is a prerequisite for the establishment of a receptive microenvironment and collagen crosslinking at distant sites, including the lung. As previously mentioned, HIF-1α and LOX are both overexpressed in breast cancer patients.

MDA-MB-231 cells exemplify the class of triple-negative breast cancers (TNBCs), which lack expression of the estrogen receptor (ER), progesterone receptor, and human epidermal growth factor receptor 2 (HER2). There are no effective therapies for triple-negative breast cancers, which have a poor prognosis with early relapse after chemotherapy [29]. In our previous study and as shown in Supplemental Fig 1, the highly metastatic breast cancer cell line MDA-MD-231 released significantly more ATP than the less metastatic MCF-7 cell line or normal epithelial cells in both normoxia and hypoxia. In addition, MDA-MB-231 cells showed higher activity of P2Y_2_R and a greater ability to invade into the ECM compared with MCF-7 cells. Thus, we hypothesized that P2Y_2_R activation by ATP released from MDA-MB-231 cells under hypoxia promotes pre-metastatic niche formation through stimulation of the HIF-1α-LOX axis. Our results showed that MDA-MB-231 cells significantly increased HIF-1α expression and LOX secretion under hypoxia; this induction of expression was abolished in the presence of apyrase, suggesting that nucleotides released from MDA-MB-231 cells in hypoxia induce HIF-1α expression and LOX secretion. These results were confirmed using ATP and UTP treatment, where ATP and UTP (10 μM) significantly induced HIF-1α expression and LOX secretion in MDA-MB-231 cells but not in MCF-7 cells. However, ATP and UTP treatment of MDA-MB-231 cells depleted of P2Y_2_R failed to increase HIF-1α expression and LOX secretion. Additionally, when we tested the ability of another highly metastatic breast cancer cell SK-BR-3 to induce HIF-1α expression and LOX release, SK-BR-3 dramatically induced HIF-1α expression and LOX release by hypoxia. In contrast, the less metastatic breast cancer cell T47D showed very weak HIF-1α expression and LOX release by hypoxia (Supplemental Fig 2A, B). These results were consistent with the ATP release level and the P2Y_2_R activity between two cells (Supplemental Fig 2C, D), suggesting that highly metastatic breast cancer cells show higher P2Y_2_R activity and induce HIF-1α expression and LOX release.

HIF-1 is a heterodimeric protein complex composed of HIF-1α and HIF-1β subunits [30]. Whereas HIF-1β expression is constitutively maintained without reference to oxygen pressure, HIF-1α expression is affected by oxygen pressure. Under normoxic conditions, HIF-1α is bound by the von Hippel-Lindau (VHL) protein, which recruits an ubiquitin ligase that targets HIF-1α for proteasomal degradation [31]. Under hypoxic conditions, prolyl hydroxylation of HIF-1α is impaired, which leads to a decrease in ubiquitination and accumulation of HIF-1α in cells [32]. However, HIF-1α expression levels are also regulated by oxygen-independent factors including nitric oxide, reactive oxygen species, growth factors, and mechanical stress [33]. LOX also induces overexpression of HIF-1α and stimulates primary tumor progression [34]. As a result, primary tumor growth leading to microenvironmental hypoxia, along with other factors, can cause changes in protein expression resulting in changes in cancer progression. According to Zhong et al. [35], epidermal growth factor-mediated activation of PI3K/PTEN/Akt/FRAP pathway was involved in the HIF-1α expression in human prostate cancer cells. Therefore, based on this report and our results, it is possible that extracellular ATP or UTP treatment might induce HIF-1α expression through P2Y_2_R activation, regardless of the oxygenation status. In case of MCF-7, it appears to induce HIF-1α level in some extent due to the inhibition of HIF-1α degradation in an oxygen-dependent manner. However, oxygen-independent mechanisms to express HIF-1α could be different from MDA-MB-231; the difference of P2Y_2_R activity in response to nucleotides between MCF-7 and MDA-MB-231 and the related signaling cascade to induce HIF-1α expression.

Moreover, our results showed that P2Y_2_R-mediated LOX secretion induced collagen crosslinking in an *in vitro* model. Hypoxia induced P2Y_2_R expression in THP-1 human monocytes, and P2Y_2_R activation by nucleotides released from MDA-MB-231 cells exposed to hypoxia stimulated cell migration and MMP induction in THP-1 cells. This result suggests that P2Y_2_R plays an important role in TAM formation and the strengthening of inflammatory conditions around tumors under hypoxic conditions. These results were then confirmed in an *in vivo* animal model. Nude mice injected with MDA-MB-231 cells showed high levels of LOX secretion, crosslinked collagen, and CD11b^+^ BMDC recruitment in the lung; however, mice that were injected with P2Y_2_R-depleted MDA-MB-231 cells did not. The inflammatory components of a developing neoplasm may include diverse inflammatory cells. For example, TAMs correlate with a poor prognosis for cancer patients [13] and produce a number of potent angiogenic and lymphangiogenic growth factors, cytokines, and proteases, all of which potentiate neoplastic progression [36]. Our data also propose that nucleotides released from metastatic cancer cells induce the recruitment of inflammatory cells including macrophages or monocytes via P2Y_2_R and that P2Y_2_R activation in monocytes mediates the secretion of MMP-2 and -9.

In regards of P2Y_2_R-mediated MMPs induction, it has been reported that P2Y_2_R-mediated transactivation of growth factor receptors leads to upregulation of several proteins including MMPs [37]. Upon activation with ATP or UTP, the Gq-coupled P2Y_2_R stimulates phospholipase C (PLC) activity which generates inositol 1,4,5 trisphosphate (IP_3_) and diacylglycerol (DAG) resulting in an elevation in [Ca^2+^]_i_ via IP_3_-dependent calcium release from intracellular stores and DAG-dependent activation of protein kinase C (PKC), respectively [38,39]. SH-3 binding domains (PXXP) within the C terminus of the P2Y_2_R bind Src to enable ATP or UTP to transactivate growth factor receptors and related adhesion focal tyrosine kinase (known as Pyk2) and downstream MAP kinases [40,41], leading to upregulation of several proteins including MMPs (Lamarca et al., 2014). P2Y_2_R can also increase the activity of MMPs; Gq-coupled P2Y_2_R activation can induce Src-dependent activation of the MMPs α-secretases [42].

**Fig.6:**
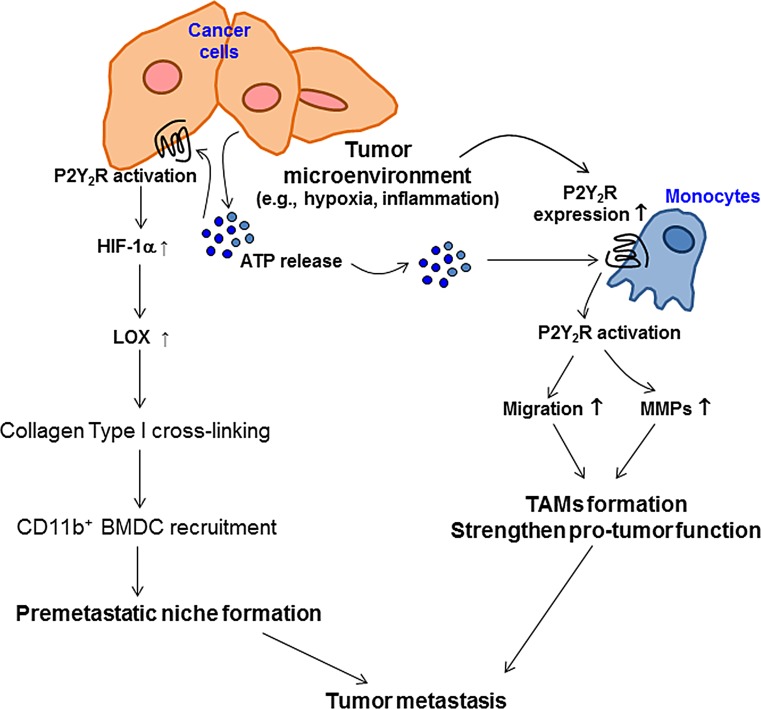
The proposed model for the role of P2Y_2_R in pre-metastatic niche formation and tumor metastasis Tumor microenvironments such as hypoxia induce ATP release from cancer cells and P2Y_2_R expression in monocytes. ATP released from cancer cells can activate P2Y_2_R both in monocytes and cancer cells. In monocytes, P2Y_2_R activation by ATP induces monocyte recruitment toward the tumor and MMPs secretion, resulting in TAMs formation and enhanced tumor growth. In addition, P2Y_2_R activation by ATP induces HIF-1α, resulting in LOX release in cancer cells. Released LOX induces collagen crosslinking, leading to the recruitment of BMDCs and pre-metastatic niche formation.

Taken together, our results demonstrate that tumor microenvironmental hypoxia induces ATP release from highly metastatic breast cancer cells and P2Y_2_R expression in monocytes. ATP released from cancer cells can activate P2Y_2_R both in monocytes and cancer cells. In monocytes, P2Y_2_R activation by ATP induces monocyte recruitment toward the tumor and MMP secretion, which promote inflammatory conditions around primary tumors. In addition, P2Y_2_R activation by ATP in breast cancer cells induces HIF-1α expression, LOX secretion, and collagen crosslinking, which forms a receptive microenvironment for pre-metastatic niche formation (Fig 6).

## MATERIALS AND METHODS

### Materials

Anti-HIF-1α and anti-LOX antibodies were purchased from Santa Cruz Biotechnology (Santa Cruz, CA). Anti-CD11b antibody was purchased from Abcam (Cambridge, UK). Enhanced chemiluminescence (ECL) Western blotting detection reagent was obtained from Amersham (Buckinghamshire, UK). 3,3%-Diaminobenzidine tetrahydrochloride (DAB) immunostaining detection reagent was obtained from Thermo (Runcorn, UK). All other chemicals, including ATP, UTP,γATP, apyrase, and β-aminopropionitrile (βAPN), were purchased from Sigma-Aldrich (St. Louis, MO).

### Cell culture

The human breast cancer cell lines MCF-7 and MDA-MB-231 were obtained from the Korea Cell Line Bank (Seoul, Korea), and the human acute monocytic leukemia cell line THP-1 was originally purchased from the American Type Culture Collection (ATCC, Vanassas, VA). The cells were grown in RPMI 1640 supplemented with 10% fetal bovine serum (FBS, Gibco-BRL), 100 IU/ml penicillin, and 10 μg/ml streptomycin (Gibco-BRL) and incubated in a humidified 5% CO_2_ incubator.

### Measurement of extracellular ATP release

Cells were incubated for 15 min at 37°C with HEPES buffer (pH 7.4) containing AOPCP, a selective inhibitor of ecto-5′-nucleotidase. The buffer was then replaced with fresh HEPES buffer containing AOPCP and incubated in 2% O_2_ at 37°C for 5 min. Supernatants were collected at specific time points, and ATP release was measured using the ENLITEN ATP assay system kit (Promega). ATP levels were calculated based on an ATP standard curve.

### Gene silencing with small interfering RNA (siRNA) or small hairpin RNA (shRNA)

Gene silencing experiments were performed with 3 independent P2Y_2_R siRNA or shRNA. Cells were transfected with 100 nM control (CTRL) siRNA or P2Y_2_R siRNA (Bioneer, Daejeon, Korea) or 10 μg/ml of shRNA (Santa Cruz; Santa Cruz, CA, USA) in serum-containing medium using Turbofect^®^ (Thermo Scientific). Gene silencing efficiency was determined by reverse transcription-polymerase chain reaction (RT-PCR) and Western blot analysis.

### RT-PCR

Total RNA was extracted using TRIzol Reagent (Invitrogen), and RT-PCR was performed using the TOPscript One-step RT PCR DryMIX (Enzynomics, Daejeon, Korea) according to the manufacturer's instructions. The primer sets used were as follows: hP2Y_2_R, 5′-GTG CTC TAC TTC CTG GCT-3′ and 5′-CTG AAG TGT TCT GCT CCT AC-3′ and hGAPDH, 5′- TCA ACA GCG ACA CCC ACT CC-3′ and 5′- TGA GGT CCA CCA CCC TGT TG-3′. Thirty cycles of amplification were performed under the following conditions: melting at 95°C for 30 sec, annealing at 56°C for 30 sec, and extension at 72°C for 30 sec.

### Western blot analysis

Cells were washed with 1× PBS, and PRO-PREP protein extraction solution was used to extract proteins (iNtRON Biotechnology, Seoul, Korea). The samples were centrifuged at 13,000 rpm for 15 min at 4°C, and the supernatants were collected for determination of the protein concentration using the Bradford method. Aliquots of 40 μg of protein were subjected to 7.5-12.5% sodium dodecyl sulfate-polyacrylamide gel electrophoresis (SDS-PAGE) for 2 h at 100 V. Proteins in conditioned media (CM) were concentrated 20-fold with Pierce concentrator 7 ml/9K, MWCO devices (Thermo, Runcorn, UK). Separated proteins in the SDS-polyacrylamide gel were transferred onto Hybond-P+ polyvinylidene difluoride membranes (Amersham). The membranes were blocked with 5% nonfat milk in Tris-buffered saline containing 0.05% Tween-20 (TBS-T) for 2 h at room temperature and then incubated with the indicated primary antibodies. The bound antibodies were detected with horseradish peroxidase-conjugated secondary antibodies and an ECL Western blotting detection reagent (Bionote, Gyeonggi-do, Korea).

### Cell migration assays

CTRL siRNA- or P2Y_2_R siRNA-transfected THP-1 cells (2 × 10^5^ cells) were added to the upper chambers of a Transwell (8 μm pore size, Corning^®^; Corning, NY), which was placed into a 24-well plate, and 500 μl of media (hypoxic CM or ATP (10 μM)- or UTP (10 μM)-containing media) was added to the lower chambers. Hypoxic CM were collected from MDA-MB-231 cells incubated in a hypoxic chamber (2% O_2_, 37°C) for 8 h in the presence or absence of apyrase (5 U/ml). After THP-1 cells were allowed to migrate for 6 h, the cells on the lower part of the insert membranes were stained with 4′,6-diamidino-2-phenylindole (DAPI) and counted in a square 500 × 500 μm field under an Olympus microscope (CKX41) equipped with a camera (Nikon, DS-U3). Three experiments were independently performed in triplicate.

### Gelatin zymography

CM were prepared from THP-1 cells or P2Y_2_R siRNA-transfected THP-1 cells treated with ATP (10 μM) or UTP (10 μM) for 24 h. The activity of MMPs was measured with gelatin zymography. Protein in the CM was precipitated with 80% cold acetone. Precipitated proteins were mixed with sample buffer (0.03% bromophenol blue, 0.4 M Tris-HCl pH 7.4, 20% glycerol, 5% SDS) and separated on 8% SDS-polyacrylamide gels containing gelatin (1 mg/ml). Thereafter, gels were washed with renaturing buffer (2.5% Triton X-100) for 1 h and subsequently incubated for 24 h at 37°C in developing buffer (50 mM Tris, 20 mM NaCl, 5 mM CaCl_2_, 0.02% Brij35, pH 7.5). Gels were stained with 0.05% Coomassie Brilliant Blue R-250 and destained with 50% methanol and 10% acetic acid. Within the blue background, clear zones indicated MMP proteolytic activity.

### *In vitro* collagen remodeling assays

Empty vector-transfected MDA-MB-231 cells (MDA-MB-231-EV) or P2Y_2_R shRNA-transfected MDA-MB-231 cells (MDA-MB-231-P2Y_2_R-shRNA) were treated with ATP (10 μM) or UTP (10 μM) in the presence or absence of βAPN (300 μM), a LOX inhibitor, for 8 h. CM were concentrated 20 times using a Pierce concentrator 7 ml/9K, MWCO device (Thermo, Runcorn, UK). Collagen type I in 0.02 N acetic acid (4 mg/ml, 100 μl) was mixed with 100 μl of concentrated CM and 10 μl of Picrosirius Red (Sigma–Aldrich) and then incubated at 37°C in an incubator for 16 h. The collagen fibers from the gel were counted under a light microscope. Each experiment was repeated three times in triplicate.

### Animal experiments

We stably transfected MDA-MB-231 cells with an expression vector encoding a shRNA targeting P2Y_2_R (MDA-MB-231-P2Y_2_R-shRNA), as described by Jin et al. [22]. Nude mice were subcutaneously injected with MDA-MB-231-P2Y_2_R-shRNA or MDA-MB-231-EV cells (5-6 × 10^6^ cells/100 μl of cell suspension). All experiments were performed in compliance with the institutional guidelines set by the Institutional Animal Care and Use Committee at Gyeongsang National University (approval number: GLA-120208-M004). Body weights and tumor volumes were measured every 3 days, starting 7 days after injection. At the end of 60 days, the mice were sacrificed. The LOX levels from the blood of mice injected with MDA-MB-231-P2Y_2_R-shRNA or MDA-MB-231-EV cells were determined by Western blot analysis. Lungs were fixed in 10% formalin, embedded in paraffin, and cut into 5-μm-thick sections. Immunohistochemistry was performed using anti-CD11b antibody. As a control, some sections were incubated in the same solution (PBS with 0.5% Triton X-100) without the addition of the primary antibody. Picrosirius Red (Sigma–Aldrich) was used for fibrillar collagen staining. After staining with CD11b antibody and Picrosirius Red, sections were counterstained with hematoxylin. Representative areas of Picrosirius Red-stained lung sections were imaged under a polarized microscope (Olympus BX-51P).

### Data analysis

Image Master^®^ VDS (Pharmacia Biotech Inc., San Francisco, CA) was used for scanning densitometry. All results are representative of three independent experiments performed in triplicate. Significant differences within experiments were evaluated by one-way analysis of variance and the Scheffe *post-hoc* test. *P*-values less than 0.05 were treated as statistically significant.

## SUPPLEMENTARY MATERIAL AND FIGURES


