# Supplementary material for: P2Y2R activation by nucleotides released from the highly metastatic breast cancer cell contributes to pre-metastatic niche formation by mediating lysyl oxidase secretion, collagen crosslinking, and monocyte recruitment
Source: Oncotarget. 2014 Sep 2;5(19):9322–34. doi: 10.18632/oncotarget.2427 (PMC4253437; doi:10.18632/oncotarget.2427)
Supplement: Supplementary file 1 [file oncotarget-05-9322-s001.pdf]

P2Y$_2$R activation by nucleotides released from the highly metastatic breast cancer cell contributes to pre-metastatic niche formation by mediating lysyl oxidase secretion, collagen crosslinking, and monocyte recruitment

Supplementary Material

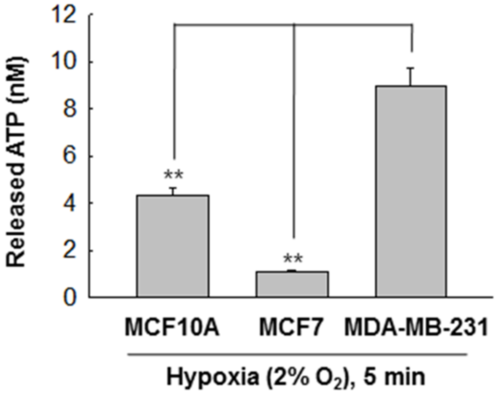

Supplemental Fig. 1: High metastatic breast cancer cell MDA-MB-231 releases high level of ATP compared to low metastatic breast cancer cell MCF-7 or normal epithelial breast cell MCF10A under hypoxic condition.

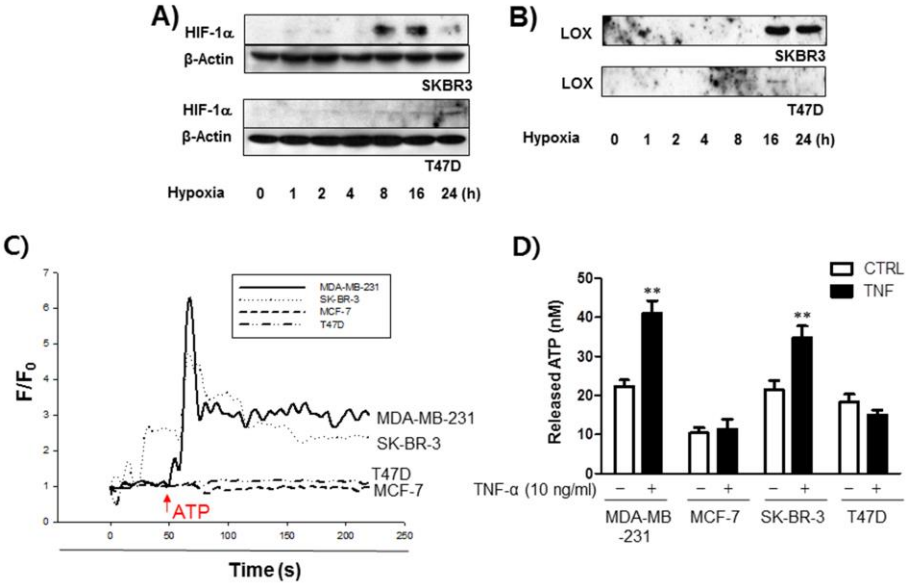

Supplemental Fig. 2: Highly metastatic breast cancer cell SK-BR-3 dramatically induced HIF-1α expression and LOX release by hypoxia. However T47D showed low ATP level and very weak HIF-1α expression and LOX release by hypoxia. In parallel, SK-BR-3 showed an elevated $[Ca^{2+}]_i$ levels in response to ATP; however T47D did not elevate $[Ca^{2+}]_i$ levels. Additionally, SK-BR-3 showed an increased ATP level like MDA-MB-231 but T47D did not.

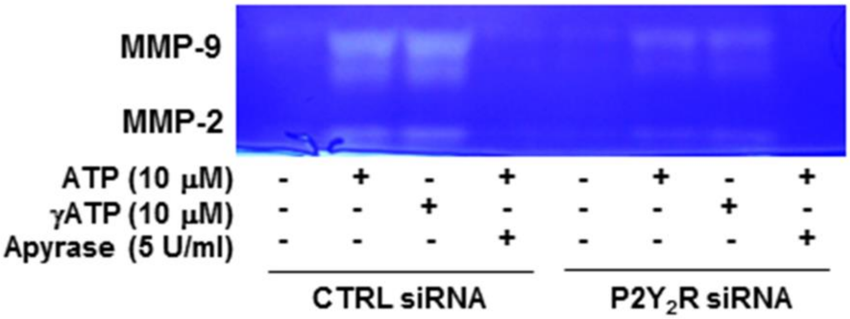

Supplemental Figure 3. ATP as well as γATP treatment prominently increased MMPs (especially MMP-9) activity in control siRNA-transfected THP-1 cells but not in $P2Y_2R$ siRNA-transfected THP-1 cells.
